# Supplementary material for: Surface-induced water crystallisation driven by precursors formed in negative pressure regions
Source: Nat Commun. 2024 Jul 26;15:6083. doi: 10.1038/s41467-024-50188-1 (PMC11282091; doi:10.1038/s41467-024-50188-1)
Supplement: Supplementary file 1 — Supplementary Information [file 41467_2024_50188_MOESM1_ESM.pdf]

Supplementary Information for "Surface-induced water crystallization driven by precursors formed in negative pressure regions"

Gang Sun[1,2] and Hajime Tanaka[3,4,*]

[1] *Social Cooperation Research Department "Frost Protection Science", Institute of Industrial Science, The University of Tokyo, 4-6-1 Komaba, Meguro-ku, Tokyo 153-8505, Japan*

[2] *Center for Advanced Quantum Studies, Department of Physics, Beijing Normal University, Beijing 100875, China*

[3] *Department of Fundamental Engineering, Institute of Industrial Science, The University of Tokyo, 4-6-1 Komaba, Meguro-ku, Tokyo 153-8505, Japan*

[4] *Research Center for Advanced Science and Technology, The University of Tokyo, 4-6-1 Komaba, Meguro-ku, Tokyo 153-8904, Japan*

* tanaka@iis.u-tokyo.ac.jp

This file includes:

Supplementary Figures 1-5

Supplementary Figures

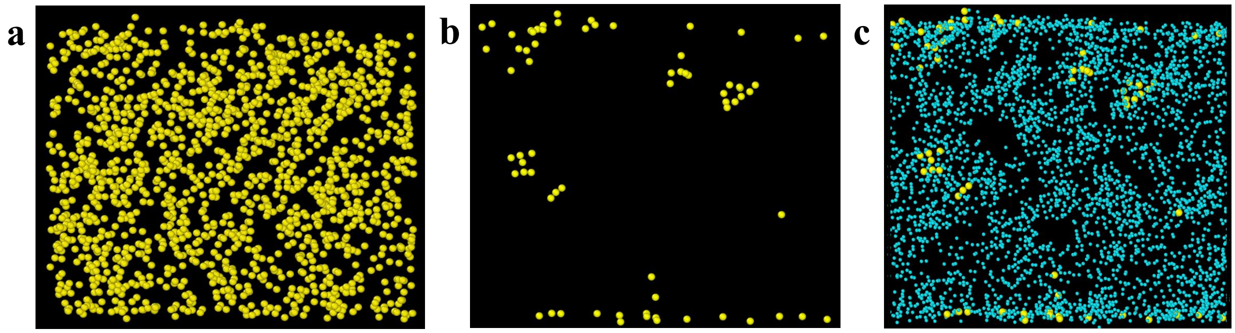

Supplementary Fig. S 1. **Preordering and ice molecules in thin water free-standing films with a thickness of** $L = 8$ **nm, relaxed at** $T = 206$ **K.** Structures at $t = 2.0$ ns: **a** Ice preorders identified by bond orientational order parameters q_6. **b** Identification of ice crystals based on the number of connections using the scalar products of the coarse-grained bond orientational order parameter Q_{12}. **c** Preordered structures identified by Q_{12}, along with the concurrent presence of ice molecules revealed by its coherency (see panel b).

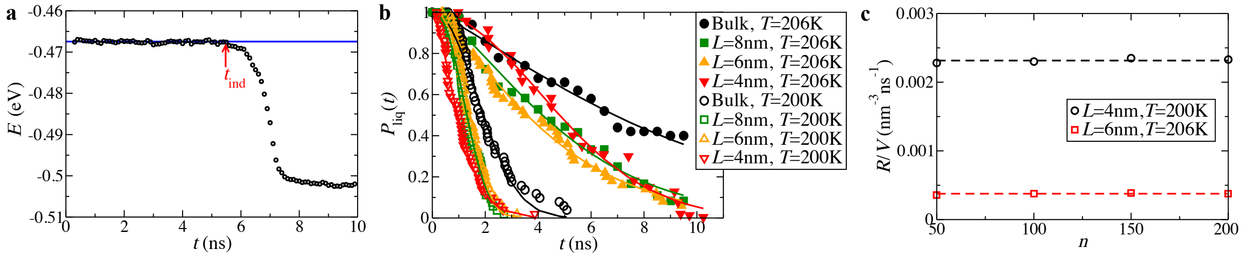

Supplementary Fig. S 2. **Estimation of the nucleation behaviour. a** Time evolution of potential energy during nucleation at T=206 K. The induction time t_{ind} is determined by tracking the temporal change in the potential energy E. **b** The probability of a system staying in a liquid state as a function of time, $P_{\mathrm{liq}}(t)$, for all the trajectories in this work. **c** The nucleation rate per volume as function of the number of statistical trajectories for two water films with thickness L=4nm and 6nm at T=200K and 206K.

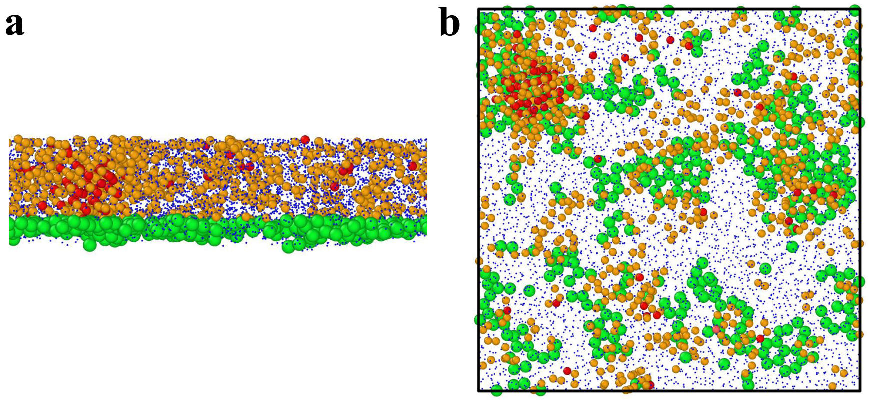

Supplementary Fig. S 3. **Spatial distribution of 2D water surface order and ice molecules near the free surface in the thin film at** $T = 206$ **K. a** Side view. **b** Top view. The highly ordered water molecules on the surface, quantified by the 2D order parameter $\overline{\lambda}_1$, are represented by large green beads. The orange and red beads denote ice preorders and ice molecules located in the interior region near the surface.

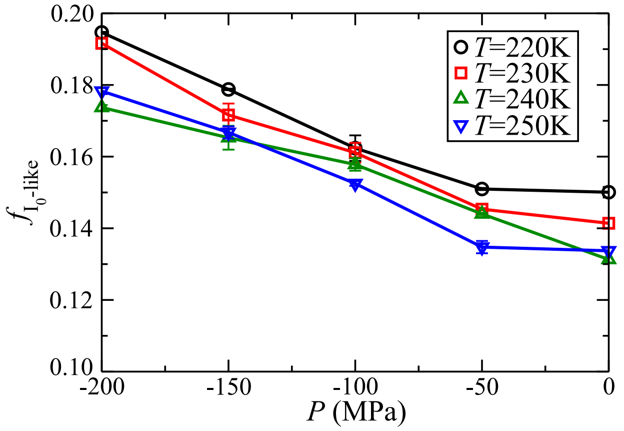

Supplementary Fig. S 4. **The pressure dependence of the fraction of Ice 0-like molecules in bulk water.** The graph illustrates the variation in the fraction of Ice 0-like molecules, $f_{I_0-\text{like}}$, as a function of pressure P at different temperatures.

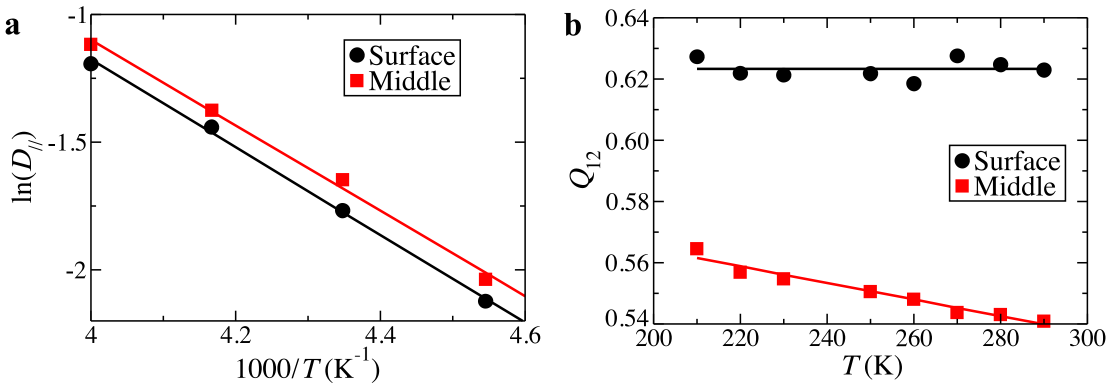

Supplementary Fig. S 5. **Surface ordering and surface mobility.** **a** The diffusion constants of molecules in the surface and middle regions of the mW water thin film plotted against temperature T. $D_{//}$ indicates the diffusion constant in the plane of the water film. **b** The structural order parameter Q_{12} in the surface and middle regions as a function of temperature T.
