# Supplementary material for: Surface-induced water crystallisation driven by precursors formed in negative pressure regions
Source: Nat Commun. 2024 Jul 26;15:6083. doi: 10.1038/s41467-024-50188-1 (PMC11282091; doi:10.1038/s41467-024-50188-1)
Supplement: Supplementary file 3 — Description of Additional Supplementary Files [file 41467_2024_50188_MOESM3_ESM.pdf]

Description of Additional Supplementary Files

Supplementary Movie S1:

Description:

Ice nucleation process in a flat water film. The ice nucleation process in a planar water film with a thickness of L=8 nm at T=206 K. Ice molecules, highlighted in yellow, and precursor molecules, indicated in cyan, are identified utilizing the order parameter Q_{12}. The free surfaces of the water film are illustrated by a grey surface mesh. The duration of this visualization spans from t=2 ns to 10 ns within a nucleation trajectory. The time interval for each frame is 0.1ns and frame speed per second in the movie is 1.

Supplementary Movie S2:

Description:

Ice nucleation process in a water droplet. The ice nucleation process in a water droplet with a radius of R = 6 nm at T = 180 K. Ice molecules, marked in yellow, and precursor molecules, highlighted in cyan, are distinguished using the order parameter Q_{12}.. Liquid molecules are represented as blue points. The temporal span of this visualization extends from t = 0 ns to 36 ns within a nucleation trajectory. Note that for the first 10 ns trajectory, the time interval for each frame is 0.2 ns, and for the rest 26 ns, it is 0.5 ns. The frame rate of the movie is 10 frames per second.
